# A computational tool for the efficient analysis of dose‐volume histograms for radiation therapy treatment plans

**DOI:** 10.1120/jacmp.v11i1.3013

**Published:** 2010-01-28

**Authors:** Anil Pyakuryal, W. Kenji Myint, Mahesh Gopalakrishnan, Sunyoung Jang, Jerilyn A. Logemann, Bharat B. Mittal

**Affiliations:** ^1^ Department of Radiation Oncology Northwestern Memorial Hospital Chicago Illinois USA; ^2^ Department of Physics University of Illinois at Chicago Chicago Illinois USA; ^3^ Department of Radiation Oncology Northwestern University Feinberg School of Medicine Chicago Illinois USA; ^4^ Department of Radiation Oncology Brown University / Rhode Island Hospital Alpert Medical School Providence Rhode Island USA; ^5^ Department of Communication Sciences and Disorders Northwestern University Evanston Illinois USA

**Keywords:** DVH analysis, IMRT, MATLAB

## Abstract

A Histogram Analysis in Radiation Therapy (HART) program was primarily developed to increase the efficiency and accuracy of dose–volume histogram (DVH) analysis of large quantities of patient data in radiation therapy research. The program was written in MATLAB to analyze patient plans exported from the treatment planning system $\left({\text{Pinnacle}}^{3}\right)$ in the American Association of Physicists in Medicine/Radiation Therapy Oncology Group (AAPM/RTOG) format. HART‐computed DVH data was validated against manually extracted data from the planning system for five head and neck cancer patients treated with the intensity‐modulated radiation therapy (IMRT) technique. HART calculated over 4000 parameters from the differential DVH (dDVH) curves for each patient in approximately 10–15 minutes. Manual extraction of this amount of data required 5 to 6 hours. The normalized root mean square deviation (NRMSD) for the HART–extracted DVH outcomes was less than 1%, or within 0.5% distance‐to‐agreement (DTA). This tool is supported with various user‐friendly options and graphical displays. Additional features include optimal polynomial modeling of DVH curves for organs, treatment plan indices (TPI) evaluation, plan‐specific outcome analysis (POA), and spatial DVH (zDVH) and dose surface histogram (DSH) analyses, respectively. HART is freely available to the radiation oncology community.

PACS numbers: 87.53.‐j; 87.53.Tf; 87.53.Xd.

## I. INTRODUCTION

Advances in imaging and radiotherapy technology have increased the complexity of radiotherapy treatment planning while improving the dose conformality to the target and dose reduction to the normal structures surrounding the target. Modern radiotherapy techniques include 3D conformal radiation therapy (CRT), intensity‐modulated radiation therapy (IMRT), and image–guided radiation therapy (IGRT). The evaluation of CRT and IMRT plans requires an in‐depth analysis of isodose distributions, dose conformality indices, normal tissue complication probabilities (NTCP), and tumor control probabilities (TCP). Conformality indices and outcome‐based plan evaluations (NTCP, TCP) are dependent on dose‐volume histogram (DVH) analysis. The clinical use of DVHs for the treatment of head and neck, breast, lung, pancreas, liver, and prostate cancers has been well reported in the literature.^(^ ^1^ ^–^ ^5^ ^)^ The sensitivity of TCP and NTCP calculations to small changes in the DVH shape points requires an accurate and efficient method of computing DVH parameters.^(6)^

A DVH represents a frequency distribution of average dose values over a 3D matrix of voxels composed of planning target volumes (PTVs) or critical structures within the patient anatomy.^(6)^ The cumulative dose–volume frequency distribution (cDVH) summarizes the simulated radiation distribution within a volume of interest in a patient from a proposed radiation therapy treatment plan. It is a useful tool for evaluating CRT and IMRT plans. The cDVH data points requested for clinical studies can be manually extracted from the commercial treatment planning system (TPS). This is reasonable for a limited number of structures and DVH data points, although the manual method is prone to errors and is time‐consuming when analyzing a large number of structures, patients and cDVH points of interest.

HART (Histogram Analysis in Radiation Therapy) program is an automated computational environment that was developed for the precise computation of dose‐volume statistics for a large quantity of patient data used for radiation therapy research. It was originally designed to expedite the extraction and analysis of DVH data from a swallowing physiology study involving 150 to 200 head and neck cancer patients treated with IMRT. Manual extraction of all the DVH data points of interest directly from the TPS was very time‐consuming and prone to error. Scripting within the planning system could also achieve the similar extraction results but would not provide the same flexibility and functionality for DVH analysis.

HART reads in the primary dose grid data from the planning system in the American Association of Physicists in Medicine/Radiation Therapy Oncology Group (AAPM/RTOG) format (Michael Goitein^(^ ^7^ ^,^ ^8^ ^)^) and accurately calculates differential and cumulative DVHs (dDVHs and cDVHs, respectively). It will query the user for arbitrary selection of DVH data points of interest and summarize the output in a simple spreadsheet format. This paper describes the development of the software (HART) and assesses its performance by comparing the results with manually extracted data from a commercial TPS. Furthermore, it also presents various aspects of treatment plan evaluation tools introduced into the program. Additional features include optimal polynomial modeling of DVH curves for organs, IMRT treatment plan indices (TPI) evaluation, spatial DVH (zDVH) and dose surface histogram (DSH) analyses, and plan‐based outcome analysis (POA) of various treatment schemes in a complete software package.

## II. MATERIALS AND METHODS

### A. Treatment protocol

This project was designed to support the analysis of an NIH‐funded, multi‐institutional study examining the swallowing function of head and neck cancer patients treated with IMRT. The ${\text{Pinnacle}}^{3}$ TPS (version 7.6c, Philips Healthcare, Best, The Netherlands) was used for IMRT planning of the patients enrolled in this study. The patients were treated using a sequential IMRT boost (SqIB) technique for three target volumes.^(^ ^9^ ^,^ ^10^ ^)^

- Planning target volume 1 (PTV1) refers to the gross tumor volume (GTV) plus the high‐ and low‐risk regional nodes with their corresponding clinical target volumes (CTVs). CTVs were defined in the region of 3 to 7 mm margin above the boundary of corresponding GTV or regional nodes. Further 3 to 5 mm margins were also added to the corresponding CTVs to draw the boundary of PTV1 region.
- Planning target volume 2 (PTV2) includes the same GTV as mentioned above, and high‐risk regional nodes and their corresponding CTVs. CTVs were defined in the region of 3 to 7 mm margin above the boundary of corresponding GTV or high‐risk regional nodes. Further 3 to 5 mm margins were added to the corresponding CTVs to draw the corresponding boundary of PTV2 region.
- Planning target volume 3 (PTV3) includes only the GTV and its corresponding CTV. CTV was defined in the region of 3 to 7 mm margin above the boundary of corresponding GTV. A 3 to 5 mm margin was added to the CTV to draw the corresponding boundary of PTV3 region.

**Figure 1:**
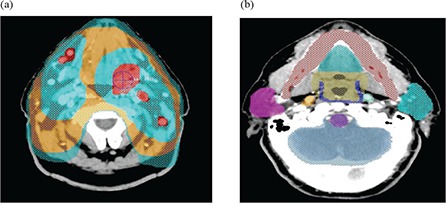
An illustration of the (a) planning target volumes: PTV1 (blue‐green), PTV2 (orange), and PTV3 (two light blue‐green sections at top) in the transverse slice, and (b) normal structures delineated using the ${\text{Pinnacle}}^{3}$ treatment planning system such as mandible (red), oral cavity (blue‐green top), oropharynx (taupe), superior pharyngeal constrictor (blue), left parotid (blue‐green right), brainstem (purple), brain (blue‐gray), and right parotid (violet).

An IMRT plan was designed for the three target volumes (PTV1, PTV2, and PTV3). A typical IMRT plan for a head and neck cancer patient is illustrated in Fig. 1. There is also a composite plan (COMPOSITE) tracking the cumulative dose of all three plans. The prescribed doses (PDs) were 3900 cGy for PTV1, 1200 cGy–1500 cGy for PTV2, and 1800 cGy–2100 cGy for PTV3, respectively. The dose fractionation was 150 cGy given twice a day on a week‐on/week‐off basis as per the institutional protocol approved by IRB. In addition to the target volumes, 24 critical structures were contoured for each head and neck cancer patient as listed in Table 1.

**Table 1:** Critical structures listed in a head and neck study.

1. Brainstem	13. Carotid vessel (left and right)
2. Brain	14. Cochlea (left and right)
3. Hyoid bone	15. Optic nerves (left and right)
4. Spinal cord	16. Eyes (left and right)
5. Oral cavity	17. Parotid (left and right)
6. Mandible	18. Base of tongue
7. Optic chiasm	19. Supraglottic larynx
8. Lips	20. Postcricioid esophagus
9. Larynx	21. Cervicothoracic esophagus
10. Oropharynx	22. Submandibular glands (left and right)
11. Glottic larynx	23. Pharyngeal constrictor (superior, middle, inferior)
12. Pituitary gland	24. Combined constrictors

Similar planning criteria were also employed in designing PTV1, PTV2, PTV3 IMRT plans and a four field PELVIS plan for ten prostate cancer patients. The three planning target volumes (PTV1, PTV2, and PTV3) were designed for IMRT boost in prostate gland during the treatment. The prescribed doses (PDs) were 900 cGy–1440 cGy for PTV1, 900 cGy–1080 cGy for PTV2, 360 cGy–800 cGy for PTV3, and 4500 cGy for PELVIS plan, respectively. The dose fractionation was 180 cGy given twice a day on a week‐on/week‐off basis as per the institutional protocol approved by IRB. Rectum and bladder were the major critical organs contoured in the IMRT plans for the prostate patients.

### B. HART format

HART computation was performed on a PC with a processor speed of 1.8 GHz, 1024 MB of RAM, and Pentium (R) 4 CPU using MATLAB (version 7.5). It uses the advanced graphical features and simulation systems available in MATLAB (Mathworks Inc., Natick, MA). MATLAB provides a flexible platform to set up a computational and graphical environment for other secondary software such as HART, Computational Environment in Radiotherapy Research (CERR), and radio‐biological outcome evaluation tool (TCP_NTCP_CALC). It offers the following major advantages over other similar commercial high level systems: (a) syntax flexibility, (b) convenient user interface, (c) user‐friendliness, (d) advanced simulation features, (e) fast algorithm development, (f) compatibility with running networks and various operating systems, (g) multidimensional array computation, and (h) an economical software. HART formats RTOG data file information and stores them in simpler text file formats in the MATLAB platform as objects, groups, and classes.

HART prompts the user to select the name of the patient and the corresponding Pinnacle exported AAPM/RTOG data files for a complete IMRT or CRT plan. MATLAB‐based codes separate each plan, the header files with target and structure identifications, and the corresponding dose grid data systematically from the AAPM/RTOG data files. This information is stored in HART formats of numerical and string arrays. The corresponding structure identifier information is stored in string or character array formats.

The software reads the spatial coordinates in 1 mm or higher resolution of a region of interest (ROI) contoured in the specific type of images (such as computed tomography (CT) scan, fused positron emission tomographic (PET) / CT images) imported into the TPS. The program also computes the accurate number of voxels in a 3D contour of an organ or target described in RTOG data format for a radiotherapy treatment plan. Simultaneously it also performs the appropriate sampling of dose within a given number of contours to generate the dDVH for the corresponding organ or target. The dDVH matrices are stored as double or floating point numerical array formats for all corresponding organs. All dDVH matrices are converted into cDVH matrices in suitable dose constraint formalism. User‐selected data points are automatically computed and simulated simultaneously for each point of interest using the DVH computation technique, as will be discussed in the next section. The automatically formatted DVH results are eventually stored in a spread sheet output format in a user‐designated location as shown in Table 2.

**Table 2:** Tables A–D are samples of the output format of HART‐computed dose‐volume histogram (DVH) data: (Table A) represents DVH data computed for the target volumes; (Tables B, C) show DVH data computed for the normalized and absolute volumes of normal structures receiving less or more than the prescribed dose (PD); (Table D) shows additional user‐specified DVH statistics for normal structures.

Table A. IMRT head and neck study — DVH data for target structures.										
*Patient Name*	*Trial*	*PD (cGy)*		*Min (cGy)*		*Max (cGy)*		*Mean (cGy)*		*Volume (cc)*
ABC	PTV1	3900		131.6		4352.1		4072.6		1391.5
	PTV2	1500		95.4		1650.5		1559.4		624.4
	PTV3	1950		216.3		2145.5		2027.2		308.6
	COMP	7350		95.4		8075.9		7214.0		X
*Absolute Volume (cc)*										
	*Structure*	<0.93PD		<0.95PD		>1.05PD		>1.10PD	>1.15PD	>1.20PD
ABC	PTV1	28.1		34.3		652.7		11.9	0.0	0.0
	PTV2	8.7		10.71		241.0		0.0	0.0	0.0
	PTV3	5.2		6.4		124.7		0.0	0.0	0.0
*Normalized Volume (%)*										
ABC	PTV1	2%		3%		47%		1%	0%	0%
	PTV2	1%		2%		39%		0%	0%	0%
	PTV3	2%		2%		40%		0%	0%	0%
Table B. IMRT head and neck study — Normalized volume (%) DVH data for normal structures.										
*Patient Name*	*Structure*	*Trial*		>0.25PD		>0.50PD		>0.75PD		>1.00PD
ABC	Brainstem	PTV1		77%		67%		24%		0%
	Brainstem	PTV2		55%		10%		0%		0%
	Brainstem	PTV3		30%		0%		0%		0%
	Brainstem	COMP		71%		44%		0%		0%
*Patient Name*	*Structure*	*Trial*		>0.25PD		>0.50PD		>0.75PD		>1.00PD
ABC	Cord	PTV1		94%		89%		15%		0%
	Cord	PTV2		71%		13%		0%		0%
	Cord	PTV3		56%		0%		0%		0%
	Cord	COMP		89%		60%		0%		0%
Table C. IMRT head and neck study — Absolute volume (cc) DVH data for normal structures.										
*Patient Name*	*Structure*	*Trial*		>0.25PD		>0.50PD		>0.75PD		>1.00PD
ABC	Brainstem	PTV1		24.9		21.9		7.7		0.0
	Brainstem	PTV2		18.0		3.4		0.0		0.0
	Brainstem	PTV3		9.8		0.0		0.0		0.0
	Brainstem	COMP		23.1		14.2		0.0		0.0
*Patient Name*	*Structure*	*Trial*		>0.25PD		>0.50PD		>0.75PD		>1.00PD
ABC	Cord	PTV1		21.3		20.0		3.5		0.0
	Cord	PTV2		16.0		3.0		0.0		0.0
	Cord	PTV3		12.7		0.0		0.0		0.0
	Cord	COMP		20.2		13.6		0.0		0.0
Table D. IMRT head and neck study — Min, Max, Mean, and Median doses and critical volume data for normal structures.^*^										
*Patient Name*	*Structure*	*Trial*	*V40*	*V50*	*V65*	*V75*	*MIN*	*MAX*	*MEAN*	*MEDIAN*
ABC	Brainstem	PTV1	0%	0%	0%	0%	280.8	3518.6	2131.8	1754.9
	Brainstem	PTV2	0%	0%	0%	0%	66.7	937.0	420.5	466.8
	Brainstem	PTV3	0%	0%	0%	0%	51.9	1025.2	337.9	510.4
	Brainstem	COMP	31%	0%	0%	0%	407.9	5155.5	2890.3	2569.7
*Patient Name*	*Structure*	*Trial*	*V40*	*V50*	*V65*	*V75*	*MIN*	*MAX*	*MEAN*	*MEDIAN*
ABC	Cord	PTV1	0%	0%	0%	0%	570.3	3211.5	2596.5	1601.4
	Cord	PTV2	0%	0%	0%	0%	26.7	927.0	511.6	461.8
	Cord	PTV3	0%	0%	0%	0%	13.0	1029.5	467.8	512.6
	Cord	COMP	50%	0%	0%	0%	603.7	5041.3	3575.9	2512.5

* V40, V50, V65, and V75 data for PTV1, 2, and 3 plans show 0% because the prescribed dose for each plan was less than 40 Gy.

**Figure 2:**
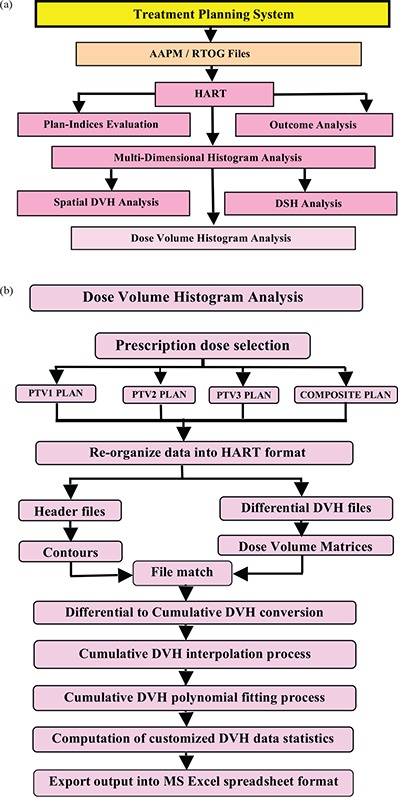
Flow chart demonstrates (a) the multiple feature selection process, and (b) the computational steps for dose‐volume histogram analysis module in HART.

The flow of information within HART is also summarized in Figs. 2(a) and (b). Figure 2(a) demonstrates the procedure to export the radiotherapy treatment plans into RTOG format of data files in the TPS. The program reads in the data files for the user‐defined analysis of the plans. Similarly Fig. 2(b) systematically presents the computational steps required for the DVH analyses of the targets and organs contoured in all plans.

### C. DVH computation and error analysis

HART reads in the treatment plan files with the AAPM/RTOG format and reorganizes the data into a simplified series of matrices. It then transforms the dDVH matrices into cDVH matrices. This is accomplished by summing up all differential voxels in a given dose range for each point of interest in the AAPM/RTOG header file. The software computes the absolute and fractional volumes of a target or critical structure receiving a dose of a certain amount using the cDVH data. The ideal dDVH for a target structure would be an infinitely narrow peak at the PD. For critical structures, this peak would ideally exist at 0 cGy. However, several peaks in a real dDVH indicate that different parts of an ROI are receiving different doses due to the local constrain and the inhomogeneous nature of the tissue.

**Figure 3:**
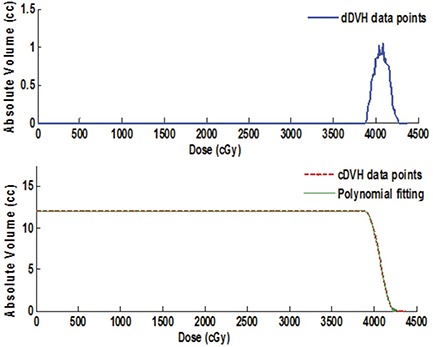
HART‐based differential (top) and cumulative (bottom) dose‐volume histograms (DVHs) simulation for a critical structure. The cumulative DVH includes an optimal polynomial approximation as shown by the dashed curve.

The smoothness of a cDVH curve depends on the bin size of the dDVH. A cosine interpolation technique with a weight factor of 0.5 was found to be a precise smoothing transition function between adjacent dose bins in the algorithm. The interpolation technique calculates the data point at the middle of two adjacent voxel elements corresponding to the adjacent dose bins in a particular ROI of the histogram. A polynomial curve fitting technique also acts simultaneously with the interpolation technique in order to find the optimal solution. The iterative piecewise polynomial fitting technique takes into account of the interpolated data point along with a series of six adjacent and nearest neighboring data points in the specified ROI of cDVH curve. This method of DVH computation employing a piecewise polynomial fitting technique and a cosine interpolation technique is the basis for the extraction of the user‐defined DVH parameters from the continuous cDVH curves in HART. This iterative process also determines optimal polynomial fitting model utilizing all cDVH data points for the corresponding target or critical structure as shown in Fig. 3. These optimal polynomial simulations for cDVH curves can also predict precise dose response models for various critical organs.

The fundamental techniques, such as normalized root mean square difference (NRMSD) and distance to agreement (DTA), were utilized to assess the HART‐computed results and the subsequent error analyses. These results were further validated by manually extracting the DVH statistics data for over 4000 specific parameters such as minimum dose, mean dose, maximum dose, and volumes coverage from the ${\text{Pinnacle}}^{3}$ system at 4 mm dose grid resolution for five head and neck protocol patients. NRMSD tests were performed for comparison of measured data from ${\text{Pinnacle}}^{3}$, and HART extracted data for absolute and normalized volumes at four different points in critical structures and six different points in targets. Similarly, DTA tests were also performed for the comparison of measured data points and HART extracted data points in the high‐dose gradient regions, specifically when the NRMSD error was greater than 1%.^(^ ^11^ ^,^ ^12^ ^)^

### D. Multi‐dimensional histogram analysis

DVHs are strong tools for three‐dimensional treatment plan evaluation. However, the drawback is the loss of spatial information. In order to achieve two‐dimensional dose distribution information across various planes and surfaces of an organ, zDVH and DSH analyses are the best evaluation techniques for CRT and IMRT plans. The zDVHs provide the spatial variation of the dose as well as the differential dose volume information along the z‐axis with respect to the CT slice positions. DSHs provide the spatial variation of the dose in a surface perpendicular to the z‐axis in the ROI of an organ.

HART accounts for all of the primary dose grid information and the 3D coordinate geometry of the target and critical structures. The algorithm generates dDVHs (secondary data) to create the corresponding cumulative DVHs, DSHs, and zDVHs. The DSHs and zDVHs help identify “hot” and “cold” regions within each slice of the target or normal structure of interest.

It should be noted that the DVH may also be obtained from $\text{zDVH}\;(D_{i},\;z)$ by summing over the z‐coordinates $z_{1},\;z_{2}\ldots z_{n}$, respectively.^(13)^ It can be expressed as,

(1) $$\text{DVH}(D_{i})=\sum_{n=1}^{N}\text{zDVH}(D_{i},Z_{n}),$$

where $D_{i}$ is a specific dose coverage in a particular CT slice at z‐coordinate position ($z_{n}$).

### E. Plan indices evaluation

High precision radiotherapy treatments such as CRT and IMRT have been frequently used in clinical routines in the past decade. In order to maintain the precision and quality of these treatments, it is essential to perform accurate patient mapping including target and organ volumes, patient immobilization, and treatment delivery utilizing an optimal treatment plan. The search for a single parameter to quantify the quality of a radiotherapy treatment plan has been ongoing but, as of yet, unsuccessful. In this perspective, a simpler method of treatment plan indices (TPI) evaluation technique of radiotherapy treatment has been incorporated into HART. A universal plan indices (UPI) set has been defined by summarizing various recognized plan indices for plan evaluation of radio‐surgical treatments, Gamma Knife (GK) treatments, and conventional LINAC‐based treatments.

The overall quality factor (QF) of a plan can also be determined by a linear combination of all plan indices in the UPI set. These indices can be assessed by utilizing the DVH statistics extracted in the HART. QF can be efficiently computed for a plan by assigning the relative weights to all UPI plan indices as a complete plan evaluation strategy. Plan indices in UPI set can be systematically described as follows:

- Target coverage index (TCI). TCI accounts for the exact coverage of PTV in a treatment plan at a given prescription dose as expressed below:
  (2) $$\text{TCI}=\frac{{\text{PTV}}_{\text{PD}}}{\text{PTV}},$$
  where ${\text{PTV}}_{\text{PD}}$ is the PTV coverage at PD, and PTV has usual meaning.
- Critical organ scoring index (COSI). COSI is a measure of both target coverage and critical organ overdose.^(14)^ It can be expressed as given below:
  (3) $$\text{COSI}=\left[1-\frac{\sum_{i=1}^{N}W_{i}V{\left({\text{OAR}}_{i}\right)}_{\geq \text{TOL}}}{\text{TCI}}\right],$$
  where $V{\left(\text{OARi}\right)}_{\geq \;\text{TOL}}$ is the fractional volume of $i^{\text{th}}$ organ at risk (OAR) receiving more than tolerance dose (TOL), and the relative weight $\left(w_{i}\right)$ of fractional volume of each organ is 1/N.
- Radiation conformity index (RCI). RCI gives a consistent method for quantifying the degree of conformity based on isodose surfaces and volumes.^(15)^ It can be expressed as:
  (4) $$\text{RCI}=\frac{{\text{PTV}}_{\text{PD}}}{{\text{PTV}}_{0.95\text{PD}}},$$
  where ${\text{PTV}}_{0.95\text{PD}}$ is the PTV coverage at 95% of PD.
- Prescription isodose target volume conformal index (PITV). PITV assesses the conformity of a treatment plan.^(16)^ However it may not be an exact parameter to identify the beam isocenter that causes a plan not to conform to the shape of the target volume in a radio‐surgery treatment. PITV can be expressed as:
  (5) $$\text{PITV}=\frac{\text{PIV}}{\text{PTV}},$$
  where PIV is the prescription isodose volume coverage for the target and normal tissues. PITV>1 and PITV<1 refers to the over treatment and under treatment regions, respectively. But it fails to account properly for the relative position of PIV with respect to PTV in radio‐surgery and LINAC‐based plans.
- Dose homogeneity index (HI). HI scales the “hot” spots in and around the planning target volumes.^(^ ^17^ ^,^ ^18^ ^)^ It can also be expressed as:
  (6) $$\text{HI}=\frac{D_{\max}}{\text{PD}},$$
  and modified dose homogeneity index (MHI) is defined as:^(18)^
  (7) $$\text{MHI}=\frac{D_{95}}{D_{5}},$$
  where $D_{\text{Max}}$ is the maximum dose point in PTV. Similarly $D_{95}$ and $D_{5}$ are the dose coverage at 95% and 5% volume of the PTV, respectively.
- Conformality index (CI) and conformation number (CN). CI measures the conformity of a treatment plan. CN accounts for the relative measurement of dosimetric target coverage and sparing of normal tissues in a treatment plan.^(^ ^14^ ^,^ ^19^ ^,^ ^20^ ^)^
  (8) $$\text{CN}=\frac{\text{TCI}}{\text{TR}},\;\text{and}\;\text{CI}=\frac{1}{\text{TR}},$$
  where treatment volume ratio (TR) is defined as:^(15)^
  (9) $$\text{TR}=\frac{\text{PIV}}{{\text{PTV}}_{\text{PD}}},$$
- Target volume ratio (TVR). TVR is an objective measure of how well the prescription isodose line conforms to the size and shape of the planning target volume.^(20)^ It is simply the inverse of ratio for PITV.
  (10) $$\text{TVR}=\frac{\text{PTV}}{\text{PIV}},$$
- Dose gradient index (DGI). It examines the steepness or shallowness of dose fall off in target volume.^(17)^ It can be expressed as:
  (11) $$\text{DGI}=\frac{{\text{PTV}}_{\text{PD}}}{{\text{PTV}}_{0.50\;\text{PD}}},$$
  where ${\text{PTV}}_{0.50\text{PD}}$ is the planning target volume coverage at 50% of PD.
- New conformity index (NCI). NCI and HI allows for the quick and simple comparison of different radio‐surgical treatment plans designed within the same or diverse radiosurgical systems, such as between LINAC and Gamma Knife.^(21)^ NCI can be expressed as:
  (12) $$\text{NCI}=\frac{\text{PIV}*\text{PTV}}{{\text{PTV}}_{\text{PD}}^{2}},$$

Thus UPI set can also be simply expressed as,

(13) $$\text{UPI}=\{X_{i}\}$$

where $X_{i}=(\text{TCI},\;\text{COSI},\;\text{RCI},\;\text{PITV},\;\text{HI},\;\text{MHI},\;\text{CN},\;\text{TVR},\;\text{DGI},\;\text{NCI})$, for a number of N major plan indices (N=10). The number (N) can be arbitrarily selected from UPI set for treatment plan evaluation in HART.

The quality factor (QF) of a treatment plan can be analytically expressed in terms of combination of above set of UPI indices as given below:

(14) $$\text{QF}=\left[2.718\exp\left(-\sum_{i=1}^{N}W_{i}X_{i}\right)\right],$$

where the values of weight factor $\left(W_{i}\right)$ can be adjusted between zero to unity for all relatively weighted indices $\left\{X_{i}\right\}$ for a user defined number of indices (N) in the UPI set. The analytical expression in the argument of the exponential function in Eq. (14) is also termed as the UPI function. Thus the fundamental application of QF and UPI evaluations is to compare the conformity of plans among various trials for a treatment.

### F. Biological modeling based outcome analysis

HART offers a convenient feature for plan‐specific outcome analysis (POA) of the radiotherapy treatments. The program employs TCP and NTCP biological modeling for the overall outcome analysis of IMRT treatments. Local TCP can be evaluated by using the Poisson statistics model, and NTCP can be evaluated by using the sigmoidal dose response (SDR) model based on equivalent uniform dose (EUD) techniques proposed by JT Lyman.^(3)^

The necessary and sufficient conditions for a TCP model require the killing of all tumor clonogens. Assuming the heterogeneous irradiation in the tissues, the overall TCP is defined as the product of the probability of killing all clonogens in each differential volume element $\left(v_{i}\right)$ of a tumor target irradiated with a dose $\left(D_{i}\right)$ in the Poisson statistics model. Furthermore, the overall TCP takes into account of the cell survival fractions assuming the single hit mechanism of the cell damage. It can be expressed as:

(15) $$\text{TCP}={\left[\frac{1}{2}\right]}^{\sum_{i}V_{i}\exp\left[\frac{2\gamma 50(1-D_{i}/\text{TCD}50)}{\ln2}\right]},$$

where the parameters TCD50 and γ50 are the dose and normalized slope at 50% probability of tumor control in the target. HART utilizes the expression Eq. (15) in order to predict the dose response probability of the tumor target.

The probit function, $\phi \left[\frac{\text{EUD}-\text{TD}50}{\text{mTD}50}\right]$, best describes the SDR model to determine the NTCP indices for critical organs irradiated during radiotherapy treatments,^(^ ^22^ ^,^ ^23^ ^)^ as expressed below:

(16) $$\text{NTCP}=\phi \left[\frac{\text{EUD}-\text{TD}50}{m\;\text{TD}50}\right]$$

The EUD or the generalized mean dose (GMD) represents the dose that, if delivered uniformly to the normal tissues or to the entire critical structure, would produce the same effect as the heterogeneous dose distribution as specified by the DVH. The parameter *m* controls the slope of the dose response curve, and TD50 determines the position of a dose response curve at 50% chance of complication in the critical structure. Furthermore, GMD can be expressed as:

(17) $$\text{GMD}={\left({\sum_{i}v_{i}D_{i}}^{1/n}\right)}^{n},$$

where *n* determines the dose‐volume dependence of a tissue which is deterministic for differences in tissue architecture. The expression Eq. (16) is implemented into HART to predict the NTCPs for the neighboring structures of the target in a specific treatment plan.

The above method, as proposed by Lyman for DVH reduction to a single dose GMD irradiated to an entire volume of an organ, is analogous to the Kutcher‐Burman (KB) reduction scheme for a non‐uniform DVH to a uniform one with an effective volume, and a reference dose equal to the maximum dose delivered to the organ.^(23)^ The KB and Lyman methods for DVH reduction schemes are found to be more consistent with the expected biological effects. These are the most robust techniques out of many available DVH reduction schemes.^(^ ^22^ ^,^ ^23^ ^)^

## III. RESULTS

Manual extraction of over 4000 DVH points of interest requires 5–6 hours for each patient plan. HART accomplished the same number of computations and simulations in 10–15 minutes. Table 2 illustrates a sample of the output computed by HART, which is exported into a Microsoft Excel spreadsheet. The DVH data and statistics specified within these sample tables were user‐defined and customized for the swallowing physiology study. Although the actual output files contain DVH data on all normal structures contoured within the treatment plan, Table 2 shows the data for only two of the contours and treatment target volumes.

Furthermore, features such as optimal polynomial fittings of cDVH curves for organs, TPI evaluation techniques, and POA options have been incorporated into HART. It has also been designed to perform various derivatives of DVH analysis such as zDVH and DSH analyses of the treatment plans. This information would be useful for the microscopic and macroscopic study of organ complication and local TCP following radiation therapy.

## IV. DISCUSSION

### A. HART validation

**Figure 4:**
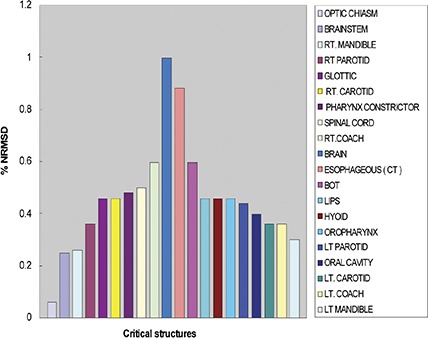
Evaluation of the normalized root mean square deviations (NRMSDs) between HART computation and manual extraction of dose‐volume histogram (DVH) data for five head and neck cancer patients.

**Figure 5:**
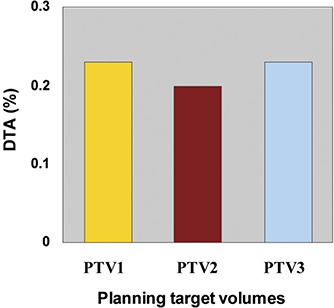
Distance to agreement (DTA) analyses for planning target volume (PTV1, PTV2, and PTV3) dose‐volume histogram data points in regions of high dose gradients. DTA is normalized relative to the prescription doses of individual plans.

DVH data calculated by HART fell within an agreement of 1% NRMSD or 0.5% DTA with the manually extracted data. Figure 4 shows the NRMSD for twenty normal structures examined within our head and neck cancer study. The DTA was also used when DVH calculation points were in high‐dose gradient regions. It was evaluated at 50% of the target and critical structure volume and normalized to the PD. Figure 5 shows the DTA for the target structures. We found that HART computations agree well with data manually extracted from ${\text{Pinnacle}}^{3}$.

The accuracy of a DVH depends on the accuracy of the dose calculation, interpolation of dose matrices, and regression of the localized fittings. The dDVH computed in ${\text{Pinnacle}}^{3}$ is dependent on the dose–grid resolution selected for a particular treatment plan. HART automatically adjusts to the ${\text{Pinnacle}}^{3}$‐based dDVH bin size for the interpolation of data points. An optimized polynomial equation performs a precise estimation of an exact number of voxels corresponding to a given dose in the continuous DVH curve in HART.

NRMSD was calculated with a limited number of data points extracted from the cDVH curves. It should also be noted that the greatest errors were observed in the regions of high‐dose gradients due to lack of exact DVH statistics estimation methodology. But the data points estimated using the optimal polynomial equations determined for the cDVH curves were found to be in good agreement with the ${\text{Pinnacle}}^{3}$‐extracted cDVH data points, especially in high‐dose gradient regions. This technique was efficient for precise computation of DVH statistics from cDVH curves in comparison to other conventional data interpolation techniques such as linear, cubic, or hermite interpolations.

NRMSDs were computed for two different cases by extracting cDVH data points using interpolation and optimized polynomial fitting of the same cDVH curves, assuming manually extracted cDVH data statistics in ${\text{Pinnacle}}^{3}$ as gold standard. NRMSDs evaluated for HART‐extracted cDVH data points utilizing above methods for various targets and organs have been presented in Table 3. Since the majority of the data points for standard DVH curves lie in low‐dose gradient regions, the overall NRMSD is obviously smaller than that estimated in the error analysis. Because of this noble DVH computational technique, HART could be a strong merit for the efficient and precise analysis of radiation therapy treatment plans.

**Table 3:** Normalized root mean square differences (NRMSDs) evaluated for HART extracted cumulative dose‐volume histogram (cDVH) data points utilizing interpolation and optimal polynomial fitting techniques for five head and neck cancer patients.

*Structures or targets*	*Number of data points plotted in*:		*Percentage NRMSD values in*:	
	*cDVH curves*	*high dose gradients*	*interpolation*	*polynomial fit*
PTV1	501	45‐50	1.2±0.2	0.6±0.1
PTV2	501	47‐52	1.2±0.2	0.6±0.1
PTV3	501	46‐54	0.5±0.1	0.5±0.1

### B. HART performance

Manual extraction of the DVH data was prone to approximately 5–10 human errors for each patient during the extraction of over 4000 DVH data points. The human errors were successfully eliminated by automating the extraction process using the HART program. The automated process included importing and reformatting the AAPM/RTOG data, cDVH computations, graphical illustration of dDVHs and cDVHs, and exporting the extracted data into a simple spreadsheet format. The program considerably shortened the execution time for DVH analysis. A comparison between manual extraction and HART computation of the sample DVH data is shown in Table 4.

**Table 4:** Comparison between manual extraction and HART computation

*Observables*	*HART Extraction*		*Manual Extraction*
Data Points		4000 – 4300
Time Period	10 – 15 minutes		5 – 6 hours
Human Error	0		5 – 10

**Figure 6:**
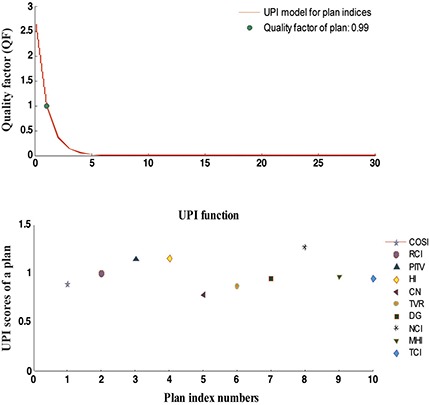
HART evaluated universal plan indices (UPI) scores utilizing ten UPI set of indices at equal relative weight $\left(W_{i}=0.10\right)$ for a prostate case treated with an IMRT boost technique. The UPI scores are presented in first simulation plot (bottom). The plot of quality factor (QF) against the UPI function is also presented as an UPI model for the given set of indices in second simulation (top). The computed value for the QF is specified in the legend of the plot. It determines the overall quality of a well designed treatment plan. Plan index numbers represent the specific number of plan indices for the identification purpose in the UPI set.

As discussed earlier, HART‐based optimal polynomial simulations for cDVH curves can also be utilized to identify precise dose response models for various critical organs in radiation therapy research. The software also offers a simple format for treatment plan indices (TPI) evaluation. UPI score in TPI functionality determines the values of individual plan indices in the UPI set. These relative scores of indices evaluate different aspects on dose homogeneity and target coverage in a treatment plan. Users have the option to select the number and type of indices to be included in the evaluation process. The ideal UPI scores for all plan indices should be unity. The overall performance of the plan indices in the UPI set can be performed by evaluating the QF for a complete plan, as discussed earlier. Typically QF equals to unity for an ideal plan, whereas the deviation from unity refers to underdose or overdose treatments in the corresponding plan. A simple demonstration for DVH statistics‐based plan evaluation technique and the quality factor assessment for a typical IMRT treatment plan designed for a prostate cancer patient has been shown in Fig. 6.

**Figure 7:**
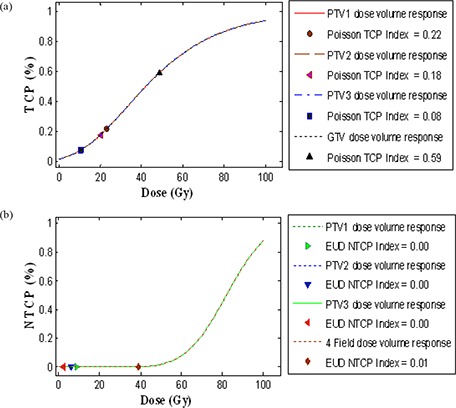
Plan specific outcome analysis (POA) of three IMRT plans and a standard four field box treatment technique for a PELVIS plan designed for a typical prostate cancer patient at T4‐stage. (a) Local tumor control probability (TCP) for planning target volumes PTV1 (prescription dose (PD) of 1260 cGy), PTV2 (PD of 1040 cGy), PTV3 (PD of 510 cGy), and for GTV (PD of 4500 cGy) with the significant control parameters reported at 95% confidence intervals: TCD50=4180 cGy, and γ50=0.6 in Poisson model is displayed on top simulation, and (b) normal tissue complication probability (NTCP) for rectum with the significant control parameters reported at 95% confidence intervals: TD50=8190 cGy, m= 0.19, n= 0.23 in Lyman‐EUD model is displayed on the bottom simulation. TCP and NTCP indices represent the corresponding probabilities of the individual treatment plans as mentioned in legend.

Furthermore, this software has also incorporated a simple and user‐friendly plan‐based outcome analysis tool to examine NTCP of critical structures and TCP in a treatment plan as discussed earlier.^(^ ^22^ ^–^ ^26^ ^)^ NTCP can be evaluated using sigmoidal dose response model (JT Lyman) and the overall TCP can be evaluated using Poisson statistics model, as displayed in Fig. 7.

### C. Multi‐dimensional histogram analysis

**Figure 8:**
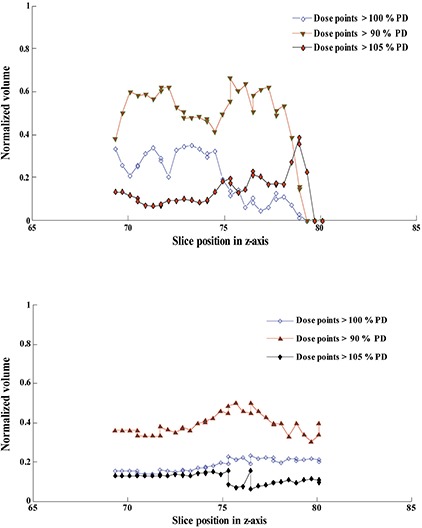
A simpler demonstration of a spatial dose‐volume histogram (zDVH) analysis (top) in a specific computed tomography (CT) slice, and a dose‐surface histogram (DSH) analysis (bottom) of a typical organ contoured in a treatment plan designed for a head and neck cancer patient. The extraction of differential dose‐volume histogram (dDVH) raw data, along with zDVH and DSH computations and simulations for 15‐20 organs contoured in a treatment plan were efficiently accomplished in 15‐20 minutes in HART.

DVHs provide statistical dose volume information and are unable to provide guidance on the location of “hot” and “cold” spots within a specific ROI. HART extracts the raw dDVH data within each CT slice, as well as gross dDVH data for normal structures contoured in a plan. So zDVH analysis can be utilized to evaluate the index of dose uniformity in such regions. Furthermore, DSH analysis is useful to study the dose distribution in serial organs such as rectum and esophagus, and parallel organs such as bladder and lungs. The results obtained from zDVH, DSH, and conventional DVH analyses would be very much useful to perform an in‐depth evaluation of treatment plans, and to study the correlation of radiation toxicity with the organ failure and local tumor control probability. Figure 8 demonstrates a simple zDVH analysis of an organ contoured in a treatment plan designed for a head and neck cancer patient.

### D. HART features

**Figure 9:**
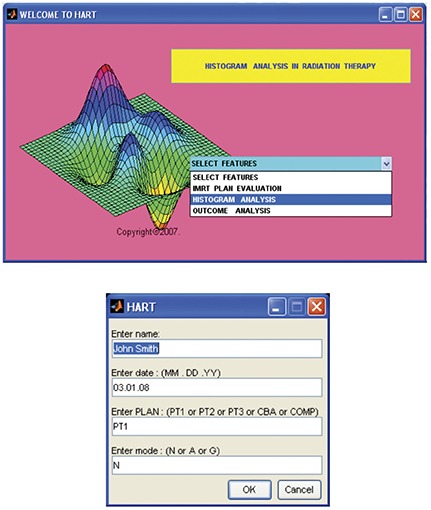
Graphical user interface demonstrates the front panel prompting selection of various features (top), and a simpler user input dialog box prompting for patient and plan information in various modes of computational operations (bottom) in HART. The automated software was designed in the MATLAB platform.

HART is a reliable and efficient tool for DVH‐based analysis in radiation therapy research. Figure 9 demonstrates a simpler format of graphical user interface of the front panel and the subsequent steps for computation in HART. It offers a flexible environment with the following functions:

- User‐defined DVH computational module
- Ability to read standardized AAPM/RTOG data file formats
- Portability in UNIX and Windows platforms
- User‐friendly graphical interface
- Structure‐specific DVH analysis options
- Customized spreadsheet output
- The dDVH data computational capability
- Optimal polynomial modeling for DVH curves
- The DSH and zDVH analysis modules
- Plan indices evaluation module
- Plan‐specific TCP/ NTCP outcome analysis tool

Since HART is not a stand‐alone executable software tool, the user needs to use MATLAB to run the program with any version of Windows or Linux operating systems. HART is also a cost‐effective and an efficient DVH analysis module. Another useful tool, CERR (Washington University, St. Louis), was developed for plan viewing and image analysis purposes but not specifically for DVH data statistics computational purposes. Because of the DVH computational limitations in CERR and other similar tools, along with the time consuming and error prone process of data extraction from ${\text{Pinnacle}}^{3}$ for a large number of patients, HART was developed as an alternative tool designed for customizable DVH data statistics computational functionality. Its application has been diversified by introducing optimal polynomial fitting model for DVH curves, TPI features, sDVH and DSH analyses, and flexible POA options of IMRT and CRT treatment plans in radiation therapy research. An illustration of statistical analysis of dose distributions in target and various critical structures utilizing HART extracted DVH data statistics for twenty head and neck cancer patients and ten prostate cancer patients has been presented in Figs. 10(a) and 10(b), respectively.

**Figure 10(a):**
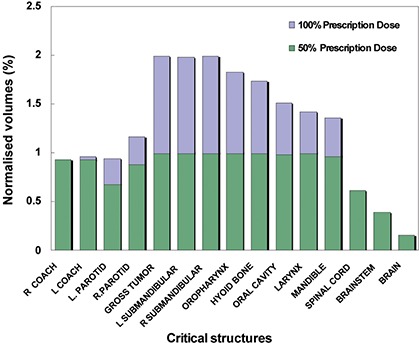
A statistical analysis of HART extracted dose‐volume histogram (DVH) data points for normalized volume coverage for a target‐gross tumor volume (GTV) and fourteen other critical structures from an IMRT treatment plan (COMPOSITE) for twenty head and neck cancer patients. DVH statistics were determined within the significance level of 95% confidence interval.

**Figure 10(b):**
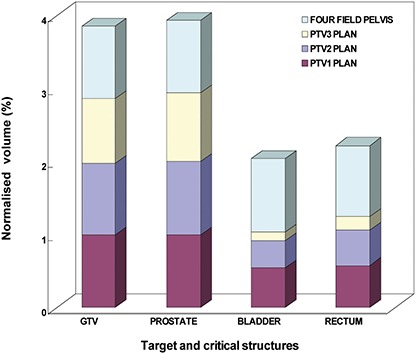
A statistical analysis of HART extracted dose volume histogram (DVH) data points for normalized volume coverage at 50% prescription dose (PD) for targets ‐ gross tumor volume (GTV) and prostate, and two major critical structures (rectum and bladder) contoured in a four field box treatment plan (PD of 4500 cGy) in combination with a series of sequential IMRT boosts (PDs of 1260×255 cGy, 1035×83 cGy, 505×100 cGy respectively) for ten prostate cancer patients. DVH statistics were determined within the significance level of 95% confidence interval.

In general, HART is a simple, user‐friendly and useful tool for summarizing and quantifying dose‐volume distributions from CRT and IMRT treatment plans. It is specifically useful in handling a large number of patient data analyses for radiotherapy research. Furthermore, by synchronizing HART with MATLAB's DICOM‐RT toolbox and other available open source tools such as CERR, we can establish a powerful platform for DVH analysis in radiation therapy research. It could also provide a convenient medium for sharing research results among radiation oncology groups. An open source environment has been established so that various users can explore HART applications to other treatment planning systems. At present, the software requires a minimal code reconfiguration for compatibility with designated treatment planning systems other than ${\text{Pinnacle}}^{3}$. Thus various users would have the privilege of using the proposed automated tool independently on a simple MATLAB environment for multipurpose analysis.

## V. CONCLUSIONS

HART is a user‐friendly, efficient, and precise DVH analysis software package built in a MATLAB environment. At present, it is also capable of importing AAPM/RTOG format files from the ${\text{Pinnacle}}^{3}$ TPS and exporting user‐defined cDVH data to a spreadsheet format. HART extracts DVH data points by utilizing noble DVH computational techniques. These results were found to be in excellent agreement with manually extracted data points from ${\text{Pinnacle}}^{3}$. Clinically, HART provides an automated system that is ideal for examining numerous DVH statistics for multiple patients and a large number of critical structures. It can also perform optimal polynomial simulations for DVH curves in order to identify accurate dose response models for various organs. Furthermore, multidimensional DVH derivative features such as zDVHs and DSHs are also available in the software package in order to pursue in‐depth evaluation of treatment plans and the analysis of treatment outcomes. It also offers a simple TPI evaluation option and a flexible POA module. The software is freely available by contacting the author. Future work includes developing additional features for multidimensional histogram analysis (xDVH, yDVH), other advanced plan evaluation tools, and statistical analysis modules with clinical relevance.

## ACKNOWLEDGEMENTS

This work was supported by the research grant entitled “Oropharyngeal Function after Radiotherapy with IMRT” from National Institute of Health / National Institute on Deafness and Other Communication Disorders (NIH/NIDCD), USA (No. RO1DC007659‐01A1).

## Supporting information

Supplementary Material Files

## References

[1] Drzymala RE , Harms WB , Purdy JA . Dose‐volume histograms for 3D radiation treatment plans. Med Phys. 1987;14(3):460.

[2] Lyman JT , Wolbarst AB . Optimization of radiation therapy, III: A method of assessing complication probabilities from dose‐volume histograms. Int J Radiat Oncol Biol Phys. 1987;13(1):103–09. 3804804 10.1016/0360-3016(87)90266-5

[3] Lyman JT . Complication probability as assessed from dose‐volume histograms. Radiat Res Suppl. 1985;8:s13–s19. 3867079

[4] Austin‐Seymour MM , Chen GT , Castro JR , et al. Dose volume histogram analysis of liver radiation tolerance. Int J Radiat Oncol Biol Phys. 1986;12(1):31–35. 3080390 10.1016/0360-3016(86)90412-8

[5] Donovan EM , Bleackley NJ , Evans PM , Reise SF , Yarnold JR . Dose‐position and dose‐volume histogram analysis of standard wedged and intensity modulated treatments in breast radiotherapy. Br J Radiol. 2002;75(900):967–73. 12515705 10.1259/bjr.75.900.750967

[6] Drzymala RE , Mohan R , Brewster L , et al. Dose‐volume historgrams. Int J Radiat Oncol Biol Phys. 1991;21(1):71–78. 2032898 10.1016/0360-3016(91)90168-4

[7] Goitein M . Specifications for tape format for exchange of planning information, version 2.2. In: Goitein M, et al., editor. Evaluation of Treatment Planning for Particle Beam Radiotherapy. Bethesda (MD): National Cancer Institute; 1985.

[8] Deasy JO , Blanco AI , Clark VH . CERR: a computational environment for radiotherapy research. Med Phys. 2003;30(5):979–85. 12773007 10.1118/1.1568978

[9] Popple R , Prellop P , Spencer S , et al. Simultaneous optimization of sequential IMRT plans. Med Phys. 2005;32(11):3257–66. 16370415 10.1118/1.2064849

[10] Dogan N , King S , Emami B , et al. Assessment of different IMRT boost delivery methods on target coverage and normal‐tissue sparing. Int J Radiat Oncol Biol Phys. 2003;57(5):1480–91. 14630288 10.1016/s0360-3016(03)01569-4

[11] Hogstrom KR , Mills MD , Meyer JA , et al. Dosimetric evaluation of a pencil‐beam algorithm for electrons employing a two‐dimensional heterogeneity correction. Int J Radiat Oncol Biol Phys. 1984;10(4):561–69. 6725043 10.1016/0360-3016(84)90036-1

[12] Low DA , Harms WB , Mutic S , Purdy JA . A technique for the quantitative evaluation of dose distributions. Med Phys. 1998;25(5):656–61. 9608475 10.1118/1.598248

[13] Cheng C , Das I . Treatment plan evaluation using dose‐volume histogram (DVH) and spatial dose‐volume histogram (zDVH). Int J Radiat Oncol Biol Phys. 1999;43(5):1143–50. 10192366 10.1016/s0360-3016(98)00492-1

[14] Menhel J , Levin D , Alezra D , Symon Z , Pfeffer R . Assessing the quality of conformal treatment planning: a new tool for quantitative comparison. Phys Med Biol. 2006;51(20):5363–75. 17019044 10.1088/0031-9155/51/20/019

[15] Knöös T , Kristensen I , Nilsson P . Volumetric and dosimetric evaluation of radiation treatment plans: radiation conformity index. Int J Radiat Oncol Biol Phys. 1998;42(5):1169–76. 9869245 10.1016/s0360-3016(98)00239-9

[16] Leung L , Chua D , Wu PM . A new tool for dose conformity evaluation of radiosurgery treatment plans. Int J Radiat Oncol Biol Phys. 1999;45(1):233–41. 10477028 10.1016/s0360-3016(99)00175-3

[17] Akpati H , Kim C , Kim B , Park T , Meek A . Unified dosimetry index (UDI): a figure of merit for ranking treatment plans. J Appl Clin Med Phys. 2008;9(3):99–108. 10.1120/jacmp.v9i3.2803 PMC5722298 18716596

[18] Yoon M , Park S , Shin D , et al. A new homogeneity index based on statistical analysis of dose‐volume histogram. J Appl Clin Med Phys. 2007;8(2):9–17. 17592460 10.1120/jacmp.v8i2.2390 PMC5722417

[19] Leung LH , Kan MW , Cheng AC , Wong WK , Yau CC . A new dose volume based plan quality index for IMRT plan comparison. Radiother Oncol. 2007;85(3):407–17. 18023487 10.1016/j.radonc.2007.10.018

[20] Paddick I . A simple scoring ratio to index the conformity of radiosurgical treatment plans. J Neurosurg. 2000;93(Suppl 3):219–22. 11143252 10.3171/jns.2000.93.supplement

[21] Collins SP , Coppa ND , Zhang Y , Collins BT , McRae DA , Jean WC . CyberKnife radiosurgery in the treatment of complex skull base tumors: analysis of treatment planning parameters. Radiat Oncol. 2006;1:46. 17173702 10.1186/1748-717X-1-46 PMC1764417

[22] Warkentin B , Stavrev P , Stavreva N , Field C , Fallone BG . A TCP‐NTCP estimation module using DVHs and known radiobiological models and parameter sets. J Appl Clin Med Phys. 2004;5(1):50–63. 15753933 10.1120/jacmp.v5i1.1970 PMC5723441

[23] Kutcher GJ , Burman C . Calculation of complication probability factors for non‐uniform normal tissue irradiation: the effective volume method. Int J Radiat Oncol Biol Phys. 1989;16(6):1623–30. 2722599 10.1016/0360-3016(89)90972-3

[24] Cheung R , Tucker SL , Dong L , Kuban D . Dose‐response for biochemical control among high‐risk prostate cancer patients after external beam radiotherapy. Int J Radiat Oncol Biol Phys. 2003;56(5):1234–40. 12873666 10.1016/s0360-3016(03)00278-5

[25] Okunieff P , Morgan D , Niemierko A , Suit HD . Radiation dose‐response of human tumors. Int J Radiat Oncol Biol Phys. 1995;32(4):1227–37. 7607946 10.1016/0360-3016(94)00475-z

[26] Luxton G , Keall PJ , King CR . A new formula for normal tissue complication probability (NTCP) as a function of equivalent uniform dose (EUD). Phys Med Biol. 2008;53(1):23–36. 18182685 10.1088/0031-9155/53/1/002

