# Supplementary figures and images for: A computational tool for the efficient analysis of dose‐volume histograms for radiation therapy treatment plans
Source: J Appl Clin Med Phys. 2010 Jan 28;11(1):137–57. doi: 10.1120/jacmp.v11i1.3013 (PMC2897015; doi:10.1120/jacmp.v11i1.3013)

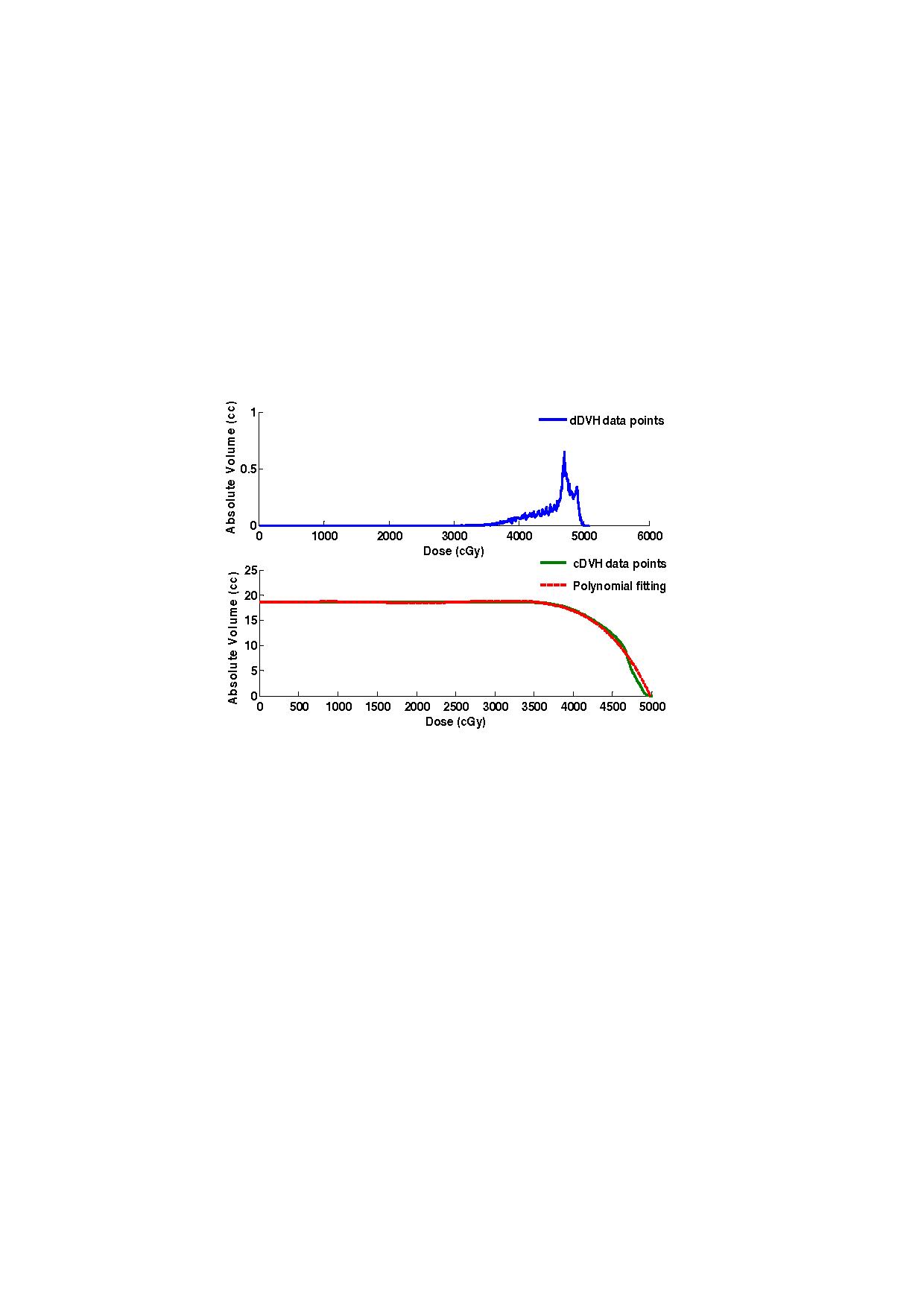

Supplement: Supplementary file 1 — Supplementary Material Files [file ACM2-11-137-s001.jpg]
